# Supplementary material for: A Simple Frailty Score Predicts Survival and Early Mortality in Systemic AL Amyloidosis
Source: Cancers (Basel). 2024 Apr 26;16(9):1689. doi: 10.3390/cancers16091689 (PMC11083900; doi:10.3390/cancers16091689)
Supplement: Supplementary file 1 [file cancers-16-01689-s001.zip › cancers-2979662-supplementary.pdf]

SUPPORTING INFORMATION

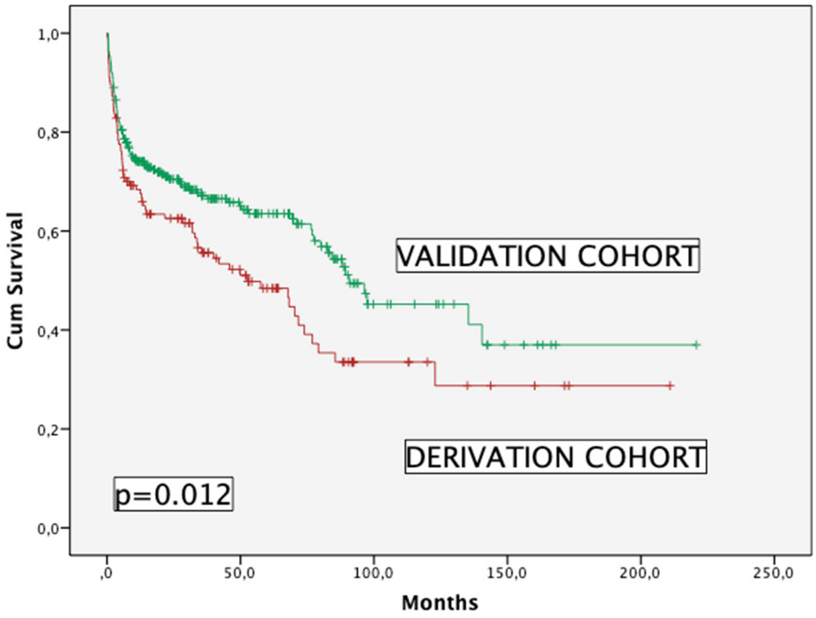


**Figure S1.** Overall survival in derivation and validation cohorts.

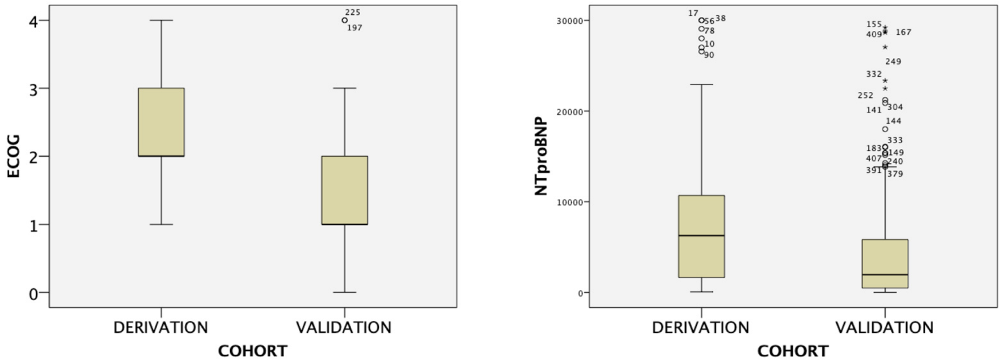


ECOG: Eastern Cooperative Oncology Group Performance Status, NT-proBNP: N-terminal pro–type-B natriuretic peptide,

**Figure S2.** Box-plot showing differences in ECOG and NTproBNP between both cohorts.

## CALIBRATION

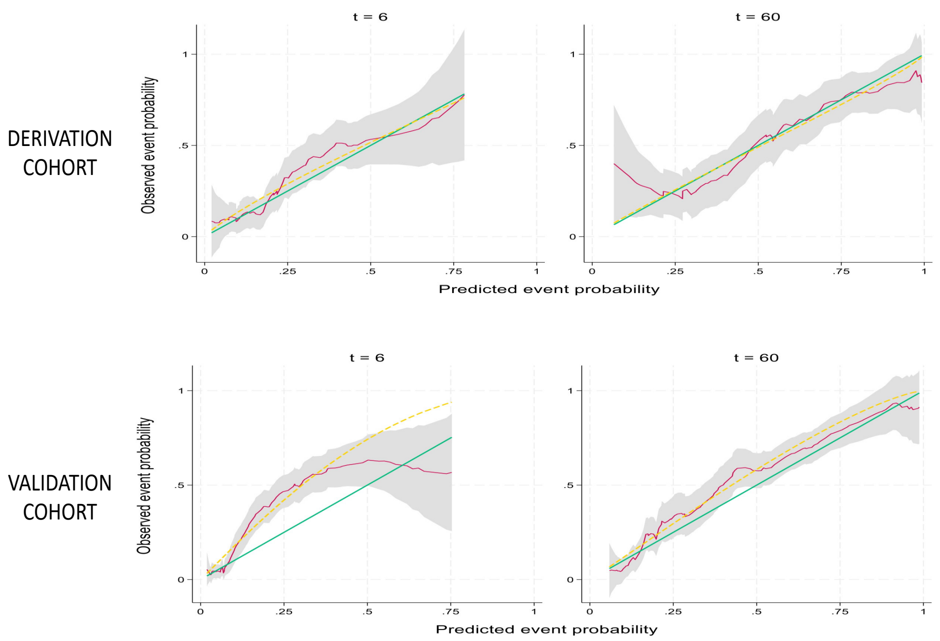


**Figure S3.** Calibration of both cohorts.

## ULTRA-FRAIL PATIENTS

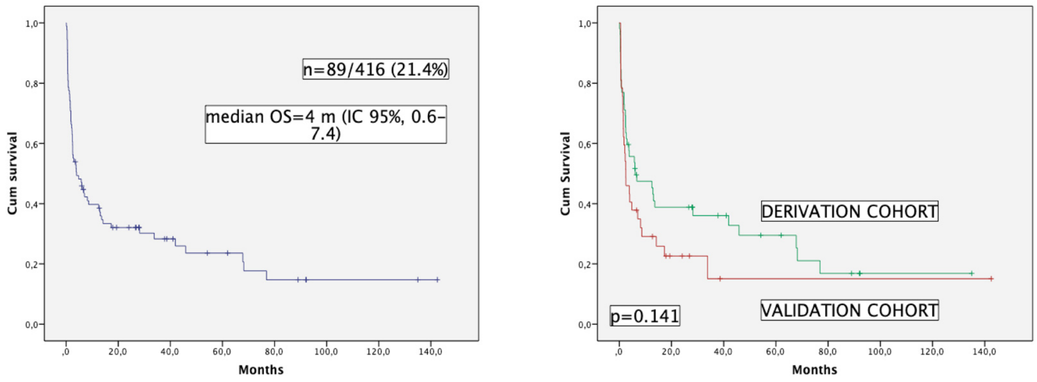


IC: Confidence Interval, mOS: median overall survival, m:months

**Figure S4.** Overall survival of ultra-frail patients.

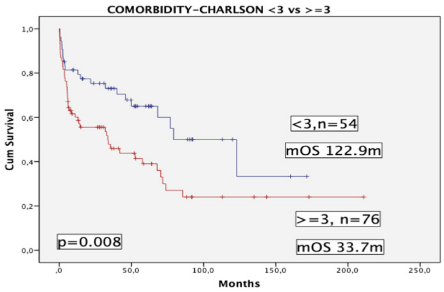


| Cox Regression Model | | | |
|---|---|---|---|
| Variables | HR | CI 95% | p |
| Age,years | 1.05 | 1.02-1.09 | 0.002 |
| ECOG, <2 vs ≥2 | 2.48 | 1.12-5.49 | 0.026 |
| NTproBNP, <8500 vs ≥8500 ng/L | 2.38 | 1.45-3.92 | 0.001 |
| CCI, <3 vs ≥3 | 1.11 | 0.93-1.32 | 0.253 |

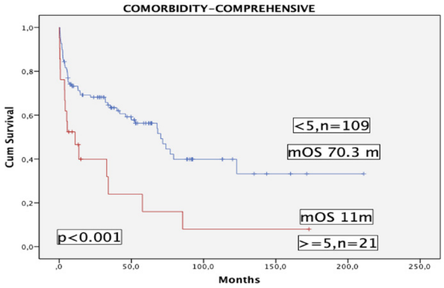


| Cox Regression Model | | | |
|---|---|---|---|
| Variables | HR | CI 95% | p |
| Age,years | 1.06 | 1.03-1.09 | <0.001 |
| ECOG, <2 vs ≥2 | 2.33 | 1.05-5.18 | 0.038 |
| NTproBNP, <8500 vs ≥8500 ng/L | 2.47 | 1.50-4.07 | <0.001 |
| Nco, <5 vs ≥5 | 1.85 | 1.01-3.40 | 0.048 |

CI: confidence interval, CCI: Charlson Comorbidity Index, ECOG: Eastern Cooperative Oncology Group Performance Status, HR: hazard ratio, Nco: number of comorbidities (comprehensive approach), NT-proBNP: N-terminal pro–type-B natriuretic peptide, m: months, mOS: median overall survival

**Figure S5.** Overall survival and Cox regression models to assess comorbidity according with Charlson Comorbidity Index (< 3 vs ≥ 3) or number of comorbidities by a comprehensive approach (< 5 vs ≥ 5).

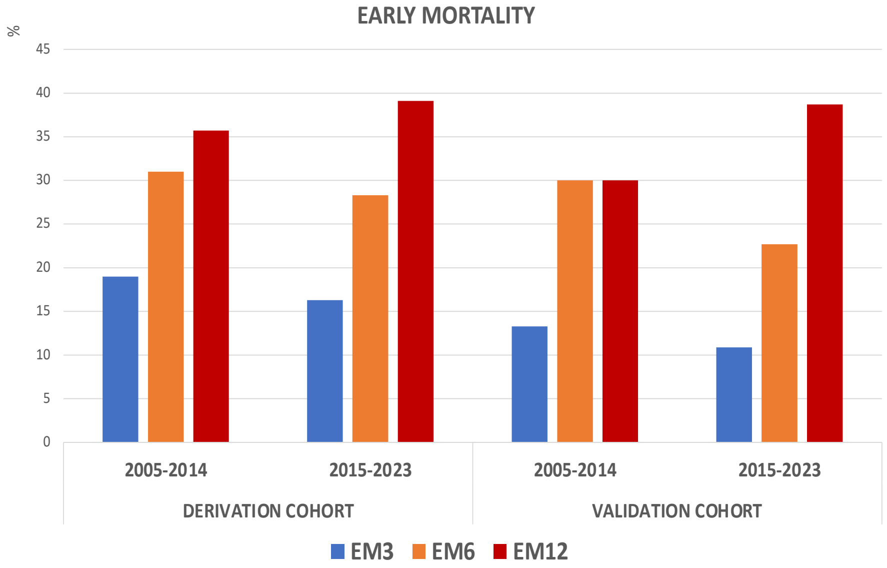


**Figure S6.** Early mortality (EM) at 3,6, and 12 months according to cohort and period of time.

**Table S1.** Percentage of comorbidities in the derivation cohort (comprehensive approach).

| COMORBIDITY | % |
|---|---|
| Hyperlipemia | 54.6 |
| Hypertension | 45.4 |
| Heart disease | 36.4 |
| Lung disease | 20.9 |
| Multiple myeloma | 19.4 |
| Type 2 diabetes | 17.8 |
| Neurological disorder | 15.5 |
| Obesity | 13.7 |
| Prior malignancy | 12.4 |
| Gastrointestinal disease | 11.1 |
| Thromboembolism | 10.9 |
| MGUS | 9.2 |
| Psychiatric disorder | 8.6 |
| Liver disease | 7.8 |
| Rheumatologic disorder | 5.4 |
| Low weight | 4.7 |
| Waldenström macroglobulinemia | 3 |
| Hepatitis B virus | 2.3 |
| Cutaneous disease | 1.6 |
| Hepatitis C virus | 0.8 |
| Splenectomy | 0.8 |
| Human immunodeficiency virus | 0 |

**Table S2.** Percentage of patients with high-risk variables in both cohorts for the three early mortality cutoffs (3, 6, and 12 months).

| | **EM3** | | **EM6** | | **EM12** | |
|---|---|---|---|---|---|---|
| | **DER** | **VAL** | **DER** | **VAL** | **DER** | **VAL** |
| **AGE ≥70** | 43.5 | 59.5 | 48.7 | 59.4 | 51 | 49.5 |
| **ECOG ≥2** | 95.6 | 70.3 | 92.3 | 62.5 | 86.3 | 50.5 |
| **NT ≥8500** | 91.3 | 62.2 | 69.2 | 43.8 | 58.8 | 35.1 |
| **Nco5** | 23.8 | | 47.6 | | 61.9 | |

DER: Derivation Cohort, ECOG PS: Eastern Cooperative Oncology Group Performance Status, EM: Early Mortality, NT-proBNP: N-terminal pro–type-B natriuretic peptide, Nco5: Five or more comorbidities, VAL: Validation Cohort
